# Parasite infections, neuroinflammation, and potential contributions of gut microbiota

**DOI:** 10.3389/fimmu.2022.1024998

**Published:** 2022-12-08

**Authors:** Jérémy Alloo, Ines Leleu, Corinne Grangette, Sylviane Pied

**Affiliations:** Center for Infection and Immunity of Lille-CIIL, Centre National de la Recherche Scientifique-CNRS UMR 9017-Institut National de la Recherche Scientifique et Médicale-Inserm U1019, Institut Pasteur de Lille, Univ. Lille, Lille, France

**Keywords:** parasitic disease, brain-inflammation, immunopathophysiology, astrocytes, microglia, gut microbiota, immunoregulation

## Abstract

Many parasitic diseases (including cerebral malaria, human African trypanosomiasis, cerebral toxoplasmosis, neurocysticercosis and neuroschistosomiasis) feature acute or chronic brain inflammation processes, which are often associated with deregulation of glial cell activity and disruption of the brain blood barrier’s intactness. The inflammatory responses of astrocytes and microglia during parasite infection are strongly influenced by a variety of environmental factors. Although it has recently been shown that the gut microbiota influences the physiology and immunomodulation of the central nervous system in neurodegenerative diseases like Alzheimer’s disease and Parkinson’s, the putative link in parasite-induced neuroinflammatory diseases has not been well characterized. Likewise, the central nervous system can influence the gut microbiota. In parasite infections, the gut microbiota is strongly perturbed and might influence the severity of the central nervous system inflammation response through changes in the production of bacterial metabolites. Here, we review the roles of astrocytes and microglial cells in the neuropathophysiological processes induced by parasite infections and their possible regulation by the gut microbiota.

## Introduction

Many of the protozoans and metazoans associated with high-mortality, high-morbidity diseases (such as *Plasmodium (P.) falciparum*, *Toxoplasma (T.) gondii* and *Trypanosoma (T.) brucei*, *Taenia (T.) solium*, and *Schistosoma (S.) mansoni*) can invade the brain and induce neuropathological disorders (1, 2). The latter are often associated with systemic or local, acute or chronic neuroinflammatory processes with a variety of clinical outcomes (**Supplementary Tables 1**, **2**). Parasite-induced brain inflammatory disorders mostly damage the central nervous system (CNS), with life-threatening consequences (3–5). In most cases, the causative neuroinflammatory mechanisms have yet to be determined. However, the pro-inflammatory cytokines and chemokines released by activated states of astrocytes and microglial cells have been identified as key factors in these neuropathophysiological processes.

Astrocytes and microglial cells are major components of the CNS, where they help to regulate homeostasis and maintain the intactness of the blood-brain barrier (BBB) (6–9). They serve as the CNS’s resident immune cells and so have a major role in the local innate immune response and inflammatory processes – particularly during pathogen invasion or tissue damage (7, 10–12). Astrocytes and microglia also function as antigen-presenting cells (13).

Deregulation of glial cell activity is commonly observed in CNS inflammatory parasitic diseases. This deregulation is often associated with the cytotoxic effects of nitric oxide, reactive oxygen species, and pro-inflammatory mediators. Nevertheless, deregulation can also be associated with a neuroprotective response (7). In fact, astrocytes secrete neuroactive molecules (including nerve growth factor, glioma-derived growth factor, ciliary neurotrophic factor, and neurotrophic factors [like leukemia inhibitory factor]) that have an important role not only in neuroregeneration but also in the attenuation of neurotoxic phenomena (13, 14). Furthermore, microglial cells can adopt an alternative anti-inflammatory phenotype characterized by the production of cytokines/chemokines that protect neurons (10) and also regulate the level of fatty acids and neurotrophic or angiogenic factors (15). Accordingly, glial cells are key regulators of pro/anti-inflammatory responses induced in the brain during parasitic diseases. These inflammatory responses are dependent on numerous parameters associated to the parasite specie, their life cycle and biology, the targeted cells, induced-immune responses, antigenic variation and immune escape strategy elicited by the parasite (16). Here, we review the various response patterns associated with acute or chronic parasite-induced brain inflammation.

During a host-parasite interaction, the inflammatory response of astrocytes and glial cells is strongly influenced by various environmental factors, including host genetic factors, immune experience, and the intestinal microbiota (11, 17). The gut microbiota (GM) is known to have a major role in the immunomodulation of the CNS in general and during neuroinflammatory and neurodegenerative diseases (such as Parkinson’s disease, Alzheimer’s disease, depression, and multiple sclerosis) in particular (17–21). We shall also review (i) the roles of astrocyte and microglial cells in the neuropathophysiological processes induced by parasite infections and (ii) how these roles are possibly influenced by the GM (12).

## Parasites that trigger acute neuroinflammation

### Cerebral malaria

**Figure 1:**
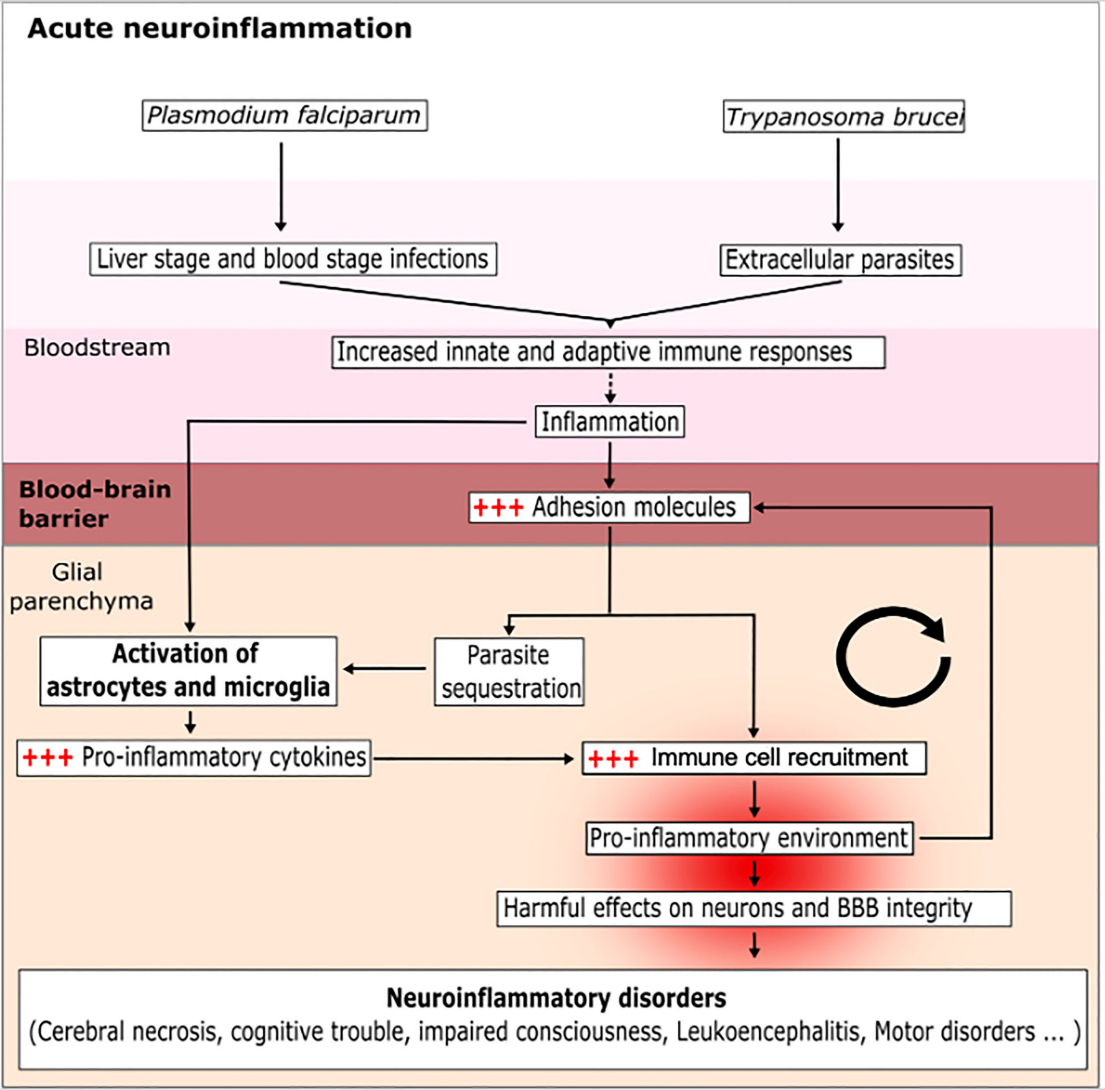
Mechanisms of the acute CNS inflammation induced by a protozoan parasite infection. The parasites *P. falciparum* and *T. brucei* enter the blood and then infect various cell types, in order to escape the immune system and splenic clearance. During this phase, the parasites activate circulating immune cells; in turn, this induces inflammation and favors the expression of adhesion molecules (ICAM-1 and VCAM-1) by endothelial cells and the activation of glial cells (astrocytes and microglia). This results in a vicious circle because the inflammation makes it easier for parasites to enter and accumulate in the glial parenchyma. The sensing of parasites by the glial cells induces the production of pro-inflammatory cytokines, allows the recruitment of immune cells and creates a pro-inflammatory environment. The brain inflammation disturbs the BBB and helps the parasite to invade the glial parenchyma. The accumulation of parasites exacerbates the activation of astrocytes and microglia cells and leads to a harmful, pro-inflammatory environment.

**Figure 2:**
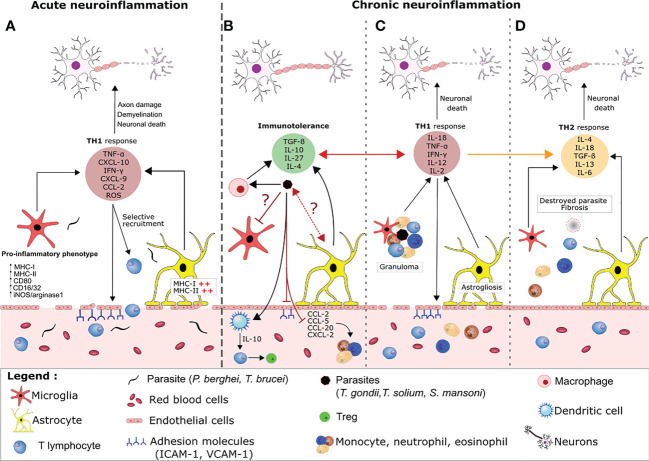
Host regulation of neuroimmune processes during acute versus chronic parasite infection. **(A)** During an acute parasite infection (e.g by *P*. *falciparum* or *T*.*brucei*), parasites cross the BBB and activate astrocytes and microglia, which produce large amounts of pro-inflammatory cytokine/chemokines (e.g CXCL-10) that recruit T lymphocytes. Together, CD8^+^ and CD4^+^ T lymphocytes favor a pro-inflammatory environment by releasing molecules like perforin, granzyme, reactive oxygen species and IFN-γ. This release leads to disruption of the BBB, which favors parasite entry, aggravates the brain inflammation, and causes collateral damage to neurons. **(B)** In contrast, immune tolerance and pro-inflammatory responses are balanced during a chronic parasite infection. The cysts release compounds that inhibit granuloma formation and the activation of resident glial cells. For example, the parasites polarize pro-inflammatory macrophages into anti-inflammatory macrophages, which suppress the production of adhesion molecules and the local TH1 response *via* TGF-β and IL-10 production. Other cyst-derived compounds polarize CD4^+^ cells into regulatory T cells (Tregs) by modulating the maturation of dendritic cells and preventing the infiltration and migration of neutrophils, eosinophils and monocytes from the peripheral system into the brain *via* the production of immunomodulatory cytokines and the blockade of chemokines and adhesion molecules. Through an as-yet unknown mechanism, the parasite also inhibits the activation of microglia and astrocytes. Nevertheless, some of the material released by the cysts elicits an inflammatory reaction (mainly characterized by the secretion of IFN-γ and TNF-α by the activated glial cells and leukocytes). The chemoattractants lead to the recruitment of neutrophils, eosinophils and monocytes, which form a granuloma. Formation of a granuloma limits the collateral damage caused by the TH1 response and enables the parasite to be contained and destroyed. The astrocytes and microglia become activated and the BBB is damaged **(C)**. Progressively, the TH1 response is replaced by a TH2 response and fibrosis occurs where the cyst was located. This fibrosis is associated with neuronal damage **(D)**.

Cerebral malaria (CM) is the deadliest complication of *Plasmodium falciparum* infection and causes approximately 500,000 deaths per year worldwide (2). Patients with CM generally develop acute neurologic manifestations: a combination of diffuse encephalopathy and decreased consciousness progresses to deep coma, seizures and, in some cases, death (22). Children who survive CM show transient or permanent neurologic sequelae and cognitive impairments (23). Along with parasite sequestration within the brain microvessels, CM is associated with an impaired immune response and excessive, uncontrolled neuroinflammation resulting from the production of pro-inflammatory cytokines and chemokines by activated astrocytes and microglial cells (3, 24, 25). We recently reported that this pro-inflammatory response is promoted by (i) the phagocytosis of infected red blood cells by microglial cells and (ii) the transfer of parasite microvesicles to astrocytes *via* microtubule-associated protein 1 light chain 3 (LC3)-dependent autophagy. The LC3-associated phagocytosis results in the production of high levels of chemokine (C-X-C motif) ligand 10 (CXCL10), chemokine (C-C motif) ligand 2 (CCL2), tumor necrosis factor alpha (TNF-α), and interferon gamma (IFN-γ)) known to be involved in the pathogenesis of CM (26, 27). As shown in **Figures 1**, **2**, these cytokines and chemokines promote disruption of the BBB and the brain infiltration of pathological αβ CD8^+^ T lymphocytes expressing chemokine (C-X-C motif) receptor 3 (CXCR3), leading to neuronal damage (25, 28–32).

### Human African trypanosomiasis

Human African trypanosomiasis (HAT) results from infection by either *T. brucei (T.b), T. gambiense*, or *T. brucei rhodesiense*. Infection by *T. brucei* starts with a hematolymphatic phase and ends with a meningoencephalitic stage. The neuropathology affected approximately 3000 people in Africa in 2015 (5) and is characterized by headaches, psychological changes, sleep disturbances, sensorimotor problems, psychiatric disturbances, and (in some cases) death (33). The parasite colonizes the brain parenchyma in several steps (34). HAT is an immune process that results from the excessive activation of perivascular macrophages, astrocytes, and microglia cells (35, 36). For example, the formation of microglial nodules and astrocytic hypertrophy has been observed in *T.b*-infected brains (10, 11). Some parasites are phagocytosed by innate immune system cells (such as perivascular macrophages) but most escape immune clearance by expressing a new variant surface glycoprotein during each humoral immune system attack (37). Activation of glial cells in the brain promotes the production of pro-inflammatory factors (TNF-α, IFN-γ, CXCL10, and CXCL9), which is associated with local functional disturbance. In *Trypanosoma*-infected brains, macrophage activation *via* the toll-like receptor 9 (TLR9) pathway and the MyD88 innate immune signal transduction adaptor (Myd88) leads to the release of TNF-α, IFN-α/ß/γ and interleukin (IL)-1ß (38). These inflammatory mediators promote the endothelial expression of intercellular cell adhesion molecule 1 (ICAM1) and vascular cell adhesion molecule 1 (VCAM1). Limited production of CXCL10 by astrocytes has also been observed but is enough to trigger the recruitment of T lymphocytes and then their infiltration into the CNS parenchyma (11). The recruited T lymphocytes are sensitized by *Trypanosoma* antigen presented by macrophages. Further massive T lymphocyte recruitment is triggered by the CXCL10 produced by activated astrocytes. In turn, this promotes the release of matrix metalloproteinase-2 and -9 (MMP2/9), which are involved in loss of the BBB’s integrity and thus create a gateway for parasite dissemination (11) (**Figures 1**, **2**).

In both CM and trypanosomiasis, the elevated production of pro-inflammatory cytokines and chemokines (by astrocytes) and the subsequent T lymphocyte recruitment are harmful. It is noteworthy that CXCL10 (produced mainly by astrocytes) might have a major neuropathogenic role; for example, CXCL10^-^/^-^ and CXCR3^-^/^-^ mice challenged with *P.berghei* ANKA or *T.brucei* control the infection more readily and are resistant to the neurologic disorder (11).

## Parasites that drive chronic neuroinflammatory processes

### Neurotoxoplasmosis

**Figure 3:**
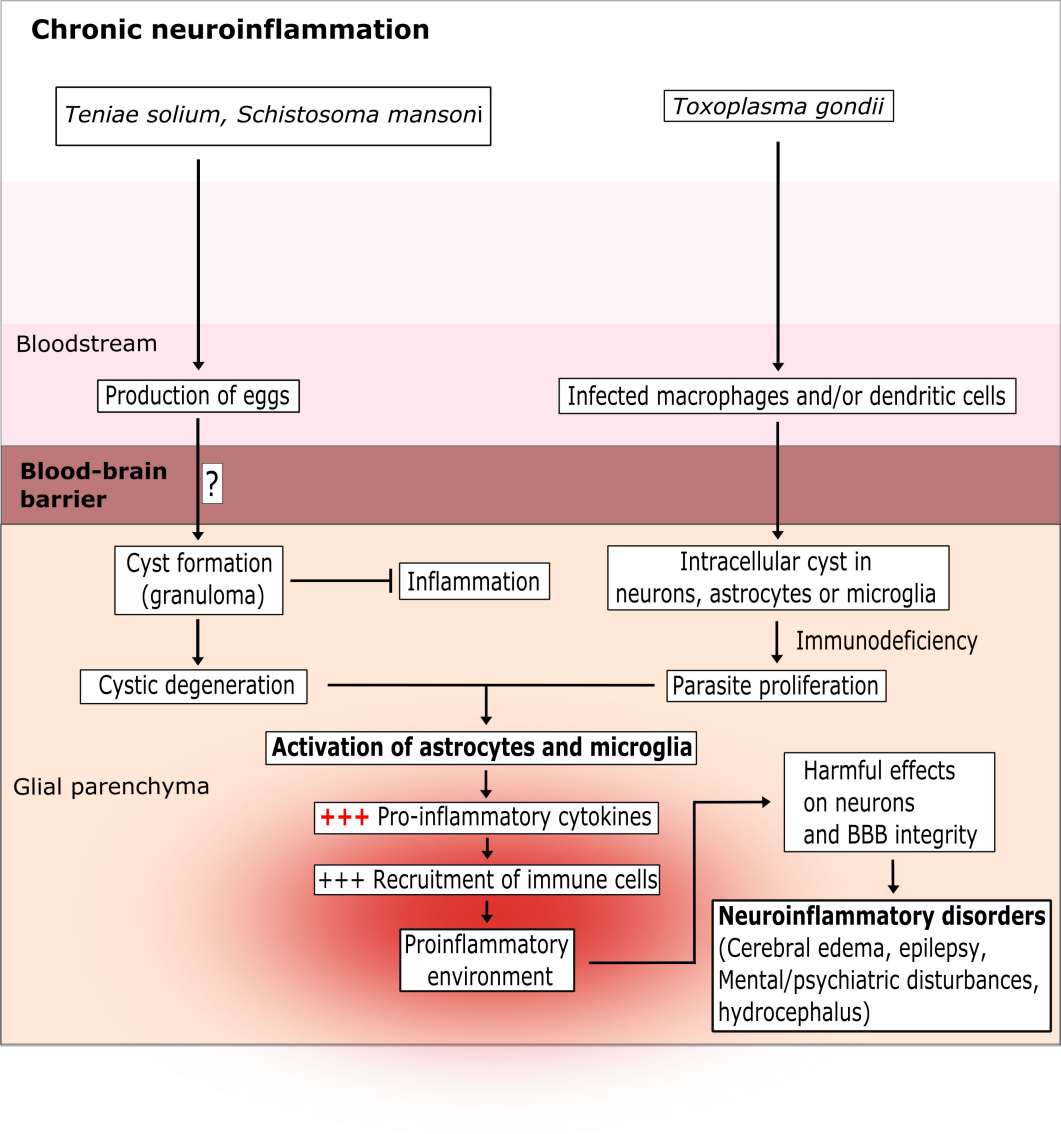
The chronic latent brain inflammation induced by parasite infection. *T. solium* and *S. mansoni* are intestinal parasites. Their eggs pass into the blood and then cross the BBB *via* as-yet unknown mechanisms. *T. gondii* infects leukocytes and crosses the BBB *via* a “trojan horse” mechanism or *via* a paracellular or transcellular route. Once inside the glial parenchyma, the parasites form extracellular cysts (for *T*. *solium* and *S*. *mansoni*) or intracellular cysts (for *T*. *gondii*) in neurons and microglia cells. The cysts can survive for several years and induce a low-level pro-inflammatory response. However, after few years or in an immunocompromised state, the cysts degenerate (for *T*. *solium* and *S*. *mansoni*) or proliferate (for *T*. *gondii*) and strongly activate astrocytes and microglia cells. In turn, this excessive activation creates a pro-inflammatory environment that damages neurons and the BBB.

Neurotoxoplasmosis is caused by the protozoan parasite *T. gondii*. The disease is contracted by ingestion of either excreted oocysts or cysts located in the muscle and nervous tissues of infected mammals (39). Toxoplasmosis is one of the world’s major food-borne diseases and is widespread in a third of the world's population (12). Primary infection with *T*. *gondii* is asymptomatic in most hosts but tends to be symptomatic in immunocompromised individuals and pregnant women. *T*. *gondii* can replicate within a wide variety of cell types, including brain cells (40). *T*. *gondii* can persist throughout the host’s lifetime in the brain as cysts, which are located principally in neurons and glial cells (39). The parasite’s initial contact with the host cells engages cell surface molecules, such as surface antigen-1, laminin, ICAM1, VCAM1, and activated leukocyte cell adhesion molecule (41, 42). The effectiveness of the human immune response to *T*. *gondii* is evidenced by low incidence of symptomatic disease, despite an overall seroprevalence of about 30%. It is clear that T cell trafficking and migration into the infected brain are critical factors in damage to the CNS. The crosstalk between *T. gondii* and the host involves a wide range of proteins and signaling networks. If an individual becomes immunocompromised, *T*. *gondii* cysts in the brain can reactivate and produce potentially lethal neurotoxoplasmosis. Activation of glial cells by interaction with *T. gondii* favors the production of pro-inflammatory cytokines and chemokines such as IL-1α, IL-6, granulocyte-macrophage colony-stimulating factor, CXCL10, CCL2, CCL3, CCL4, and IFN-γ (39, 43). Astrocytes that lack the IL-6 receptor or glial fibrillary acidic protein lose their ability to control the parasite. As a result, the inflammation spreads throughout the brain and can be fatal (44) (**Figures 2**, **3**). Excessive brain inflammation and neuronal damage are prevented by the autoregulation of astrocytes and neurons *via* the secretion of anti-inflammatory cytokines, such as IL-27 and transforming growth factor beta (TGF-β); these cytokines have immunosuppressive activity and influence T cell functions (11, 39, 43, 45).

### Neurocysticercosis

Neurocysticercosis (NCC) is a CNS disease caused by *T. solium*. It was responsible for around 28,000 deaths in 2010. NCC is a neglected tropical disease and is mainly found in countries with ineffective healthcare systems (46). The clinical manifestations associated with NCC include seizures, epilepsy, focal neurologic impairments, elevated intracranial pressure, and cognitive decline. The disease manifestations depend on the number and size of the cysticercus or the parasitic stage development (47). NCC is thought to result from the neuroimmune processes induced by the parasite’s eggs, which are produced in the intestine. Once the eggs have entered the bloodstream, they disseminate to various tissues - including the brain. After crossing the BBB, *T. solium* forms a cyst within the brain and can persist for several month or years - probably through active evasion and immunosuppression (47–49). After 3 to 10 years, the larvae degenerate (via as-yet unknown mechanisms) and lose their ability to control the CNS inflammation (47, 50). A TH1 pro-inflammatory response is then engaged; the TNF-α, IFN-γ, IL-1ß, IL-18, and IL12 released by microglia and macrophages trigger the expression of the adhesion molecules ICAM1 and VCAM1 and the chemoattractants and chemokines CXCL2, CXCL8, CCL5, CCL2, and CCL20 by astrocytes and endothelial cells. This damages the BBB and leads to cerebrospinal fluid leakage, greater leukocyte migration, and granuloma formation in the CNS (47, 50). The granulomas primarily comprise multinucleated giant cells, macrophages, T and B lymphocytes, plasma cells, neutrophils, eosinophils, and microglia around a degenerated cyst. The TH1 response is counterbalanced by a TH2 response characterized by IFN-γ, IL-18, IL-4, IL-10, IL-13 and TGF-β production and that leads to progressive fibrosis (47, 49, 51). Neuroinflammation and granuloma in NCC constitute a “double-edged sword”: the granuloma protects the adjacent CNS tissue from the parasite but leads to damaging fibrosis and seizures (52) (**Figures 2**, **3**).

### Neuroschistomiasis

Neuroschistomiasis (NS) is a poorly characterized neuroinflammatory disease remaining caused by *S. mansoni, S*. *japonicum*, and *S*. *haematobium.* The prevalence of NS has not been reliably determined, although at least 600 million people in at least 79 countries are thought to be at risk of infection (53). About 2 to 4% of individuals infected by *S.japonicum* develop neurological manifestations (54). Patients suffering from NS generally have headaches, visual disturbances, delirium, seizures, motor impairments, ataxia, and encephalopathy (55). NS results from an immune process associated with the ectopic deposition of eggs in the leptomeninges and the cerebral cortex, *via* as-yet unknown mechanisms (53). By analogy with NCC, it has been suggested that eggs in the brain escape the immune response and survive there for weeks. However, larval degeneration triggers the release of antigens and a TH1 pro-inflammatory response favoring (i) the recruitment of CD4^+^ T cells, eosinophils, macrophages and monocytes, and (ii) granuloma formation, leading to brain tissue necrosis (53). In fact, the TH1 response (characterized by IFN-γ, IL-2, IL-4, IL-5, IL-9 and IL-13 production) is gradually replaced by a TH2 anti-inflammatory response with overproduction of IL-10 (limiting the degree of neurologic damage) (54) (**Figures 1**, **2**). However, many questions remain with regard to the pathophysiological mechanisms associated with NS, including how the parasite interacts with glial cells, how it crosses the BBB, and how it survives in the brain.

Indeed, the current lack of effective treatments and the emergence of resistant parasites highlight the urgent need for novel biomarkers and potentially curative therapeutics for these neuroinflammatory diseases. Targeting the GM (in order to reduce the glial cells’ pro-inflammatory activity and thus counteract the brain inflammation) is an interesting strategy, as already demonstrated for Alzheimer’s disease, Parkinson's disease or multiple sclerosis (17, 19, 20). However, researchers are only now starting to investigate the GM’s effect on parasite-induced neuroinflammatory diseases and to develop prebiotics, probiotics or fecal transplantation techniques for preventing and dampening the disease process (56–58).

## Involvement of the GM in parasite-driven CNS inflammation

The host’s microbiota is a consortium of bacteria, viruses, fungi, archaea, and protozoa. These microorganisms co-evolved with the host and colonize the skin and the respiratory, urogenital and gastrointestinal tracts (59). Commensal communities are essential for the body’s development and functioning. The GM harbors trillions of microbes (mostly bacteria); an adult human harbors around 200 to 300 different bacterial species, most of which are located in the ileum and the colon (60) and interact with each other (61). Whereas the human genome contains about 23,000 genes, the GM provides up to 10 million genes in total (62) and about 600,000 in a given individual (63). Even though the GM is highly resilient, its composition changes and can be modulated by factors like diet, age, sex, geographic location, ethnicity, exercise, and drug intake (63). The GM’s crucial role in human health comes through a variety of physiological mechanisms: modulation of the gut barrier and the host’s metabolism (64, 65), local and systemic immune regulation (66, 67), neural development, and emotional development (65, 68–70). Abnormal changes in the GM’s composition or activity – a so-called state of dysbiosis – are thought to be involved in the development of many diseases. It has recently been shown that dysbiosis contributes to extra-intestinal diseases and notably those affecting the CNS (71). Dysbiosis is involved in the development of the brain (68) but also in mental illnesses, such as eating disorders (72), autism (73), schizophrenia, anxiety disorders, mood disorders (74), and neurodegenerative diseases associated with neuroinflammation (such as Alzheimer’s and Parkinson’s diseases) (21, 75–79). In patients suffering from neurodegenerative diseases, a growing body of evidence indicates an association between GM dysbiosis and leakage of the intestinal barrier. This leakage favors inflammatory responses and the release of compounds into the systemic circulation, which worsen the brain inflammatory process (21, 80, 81).

A link between the GM and the brain has been evidenced by experiments in germ-free mice, in which damage to the BBB can be partially restored by fecal transplants (82, 83). Microglia from germ-free mice or mice treated with an antibiotic presented an immature profile and an impaired immune response. This defect of the GM is associated with changes in microglial mRNA profiles in the germ-free mice: genes involved in cell activation, pathogen recognition, and host defense were downregulated in the animals’ microglia, whereas genes encoding survival factors (which are usually suppressed in conventional animals) were upregulated (84, 85). Furthermore, other experiments in animal models showed that antibiotic-associated dysbiosis reduced neurogenesis in the hippocampus and thereby induced memory loss (83, 86). On the same lines, many research studies have highlighted the GM’s impact on the BBB’s integrity and the activity of CNS cells – either by modulating metabolites produced by bacterial species (82, 87–91), modulating the immune response (86, 92–96) or even by influencing the activity of the vagal nerve (97).

Even if *Toxoplasma*, *Plasmodium*, *Trypanosoma* and *Schistosoma* are all known to disturb the GM, a link with brain inflammation resulting from infection is not clearly established (56, 98–101). However, modification of the GM’s composition has been described during toxoplasmosis since gram-negative bacteria like the enterobacteria aggravated the ileitis induced by *T. gondii* infection by perturbing tryptophan metabolism, dopamine level and decreasing Treg counts (66, 102, 103). Ileitis in *T. gondii-*infected mice was associated with elevated intestinal permeability, greater bacterial translocation, mild-to-moderate meningitis, behavior and cognitive disorder in wild-type mice (relative to germ-free mice) (102, 104–107).

GM dysbiosis has also been observed in *P. yoelii-* and *P.berghei* ANKA-infected C57BL/6 mice. Dysbiosis was generally associated with a low *Firmicutes* count and elevated *Proteobacteria* and *Verrucomicrobiae* counts (98, 108). These preclinical results were confirmed in a study of *P. falciparum*-infected children living in a rural village in Mali: the GM was enriched in *Bacteroidetes* and depleted in *Firmicutes*, relative to the GM in European children. We are investigating the possible association between the development of cerebral malaria and the composition of the GM. Our preliminary results suggest a role for GM dysbiosis in the brain inflammatory process leading to cerebral malaria in C57BL/6 susceptible mice infected with *P. berghei* ANKA by modifying the pro-inflammatory response of glial cells. Moreover, dysbiosis induced by antibiotics or probiotics protect against cerebral malaria These data corroborate previous published work showing that the *P. berghei* ANKA infection induced changes in the GM which in turn impact malaria severity (56, 98). GM dysbiosis has also been observed during cerebral trypanosomiasis gut microbiota in mice and human (101). GM dysbiosis was associated with alterations in fatty acids and bile acids metabolism in infected mice (109). In addition, it has been recently described that the increase of BBB permeability correlates with increased levels of IL-17, IL-22 and brain infiltration by bacterial metabolites, notably butyrate (110). This results in a rise in glutamate, excitotoxicity and cell death in the brain parenchyma (111). In neuroschistosomiasis, a recent study has shown that CNS macrophages including microglia play a role in forming granulomas around the parasite eggs (55). Indeed, in mice infected with  *S. japonicum* it has been recently shown alterations in the intestinal barrier and GM composition upon infection with a possible link to microglial cells phenotype (112, 113).

## The consequences of GM modification by a parasite infection

Parasite infections can induce dysbiosis and thus impact the host’s inflammatory response and susceptibility to infection (114). Indeed, it has been demonstrated that the GM influences the pathogenicity of certain parasites or helminths directly (through bacteria-parasite interactions) or indirectly (by providing essential metabolites and therefore a more conducive environment for parasite proliferation and growth) (88). Of note, *in vivo* imaging of *P. berghei-*expressing luciferase has shown high amount of parasites in the mesentery and intestinal lumen which perturbated GM by favouring interactions and nutritional competition with gut bacteria (98, 115–119). Parasites can also modulate the GM composition indirectly through immunoregulatory factors. It is the case for *T. gondii*, IFN-γ and NO released during infection can cause the loss of Paneth cell and inhibit the production of antimicrobial peptides (116–119).

In some cases, the GM is enriched in certain bacterial phyla that reduce the severity of the disease associated with protozoan, fungal, helminth or bacterial infections (103). This is the case for *P. berghei* ANKA infections: the GM of "resistant" mice had a higher proportion of *Firmicutes* (56). It has also been shown that mice experimentally colonized with the α-galactosyl-expressing intestinal pathobiont *E. coli* O86: B7 produced anti-α-galactosyl antibodies, which prevent invasion of the liver by sporozoites (120).

## Could modulation of the GM help to prevent the neuroinflammation induced by parasite infections?

**Figure 4:**
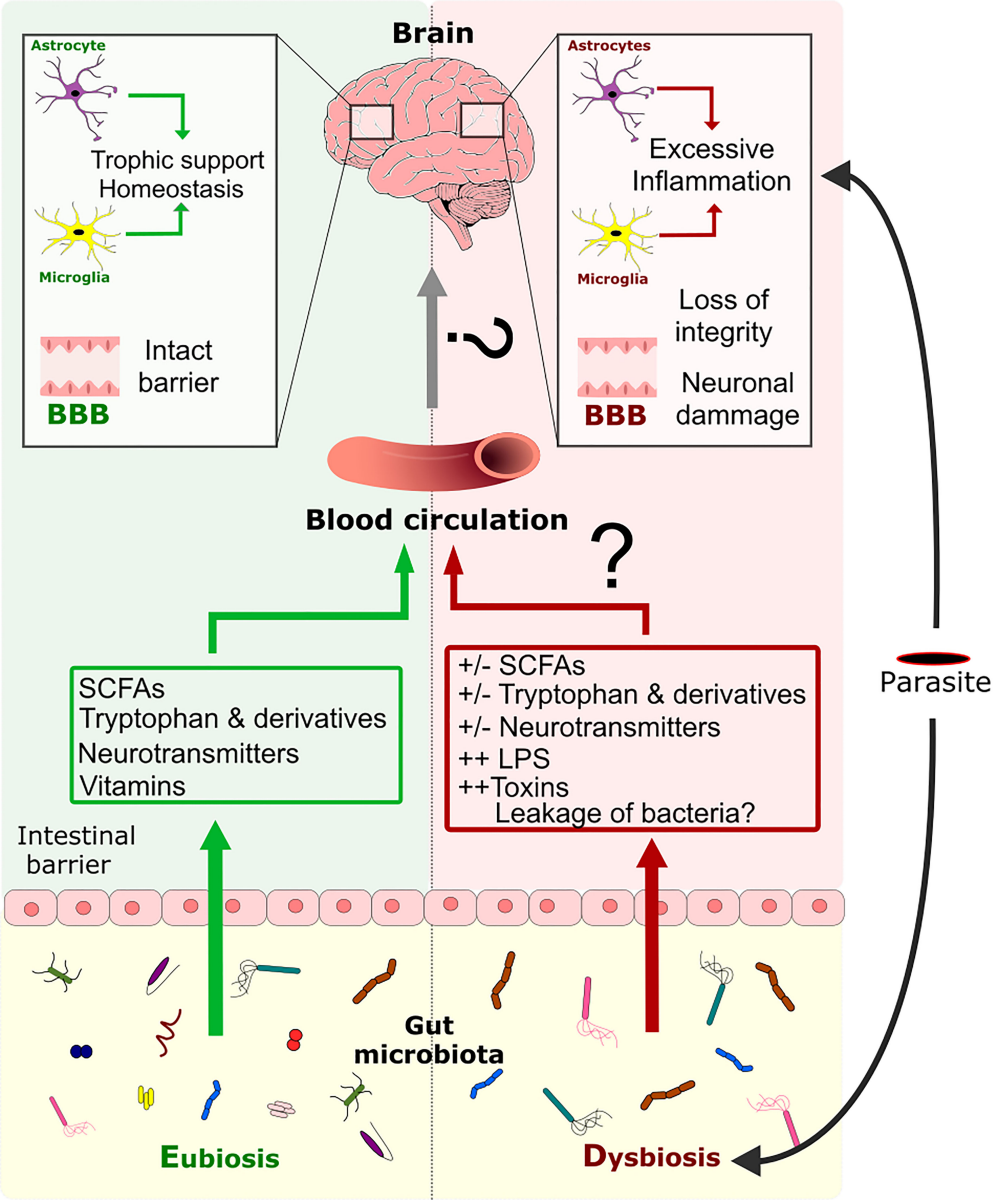
Modulation of the CNS inflammation response by the GM during a parasite infection. Eubiosis of GM favors the maintenance of the gut barrier’s intactness (122, 123). Moreover, the GM can produce metabolites like SCFAs, tryptophan, tryptophan derivatives, neurotransmitters, and vitamins, which are disseminated through the host’s circulation. These metabolites are known to have an impact on the BBB’s intactness and on CNS cells like astrocytes and microglia. Infection by a parasite induces dysbiosis of the GM directly or indirectly, which perturbs metabolite production, impairs gut barrier intactness and allows the possible translocation of bacteria throughout the organism. Dysbiosis is associated with an impairment of glial cell activity and loss of the BBB’s intactness. Dysbiosis might also favor the excessive pro-inflammatory response of glial cells induced by the parasite and that leads to neuronal damage.

**Figure 5:**
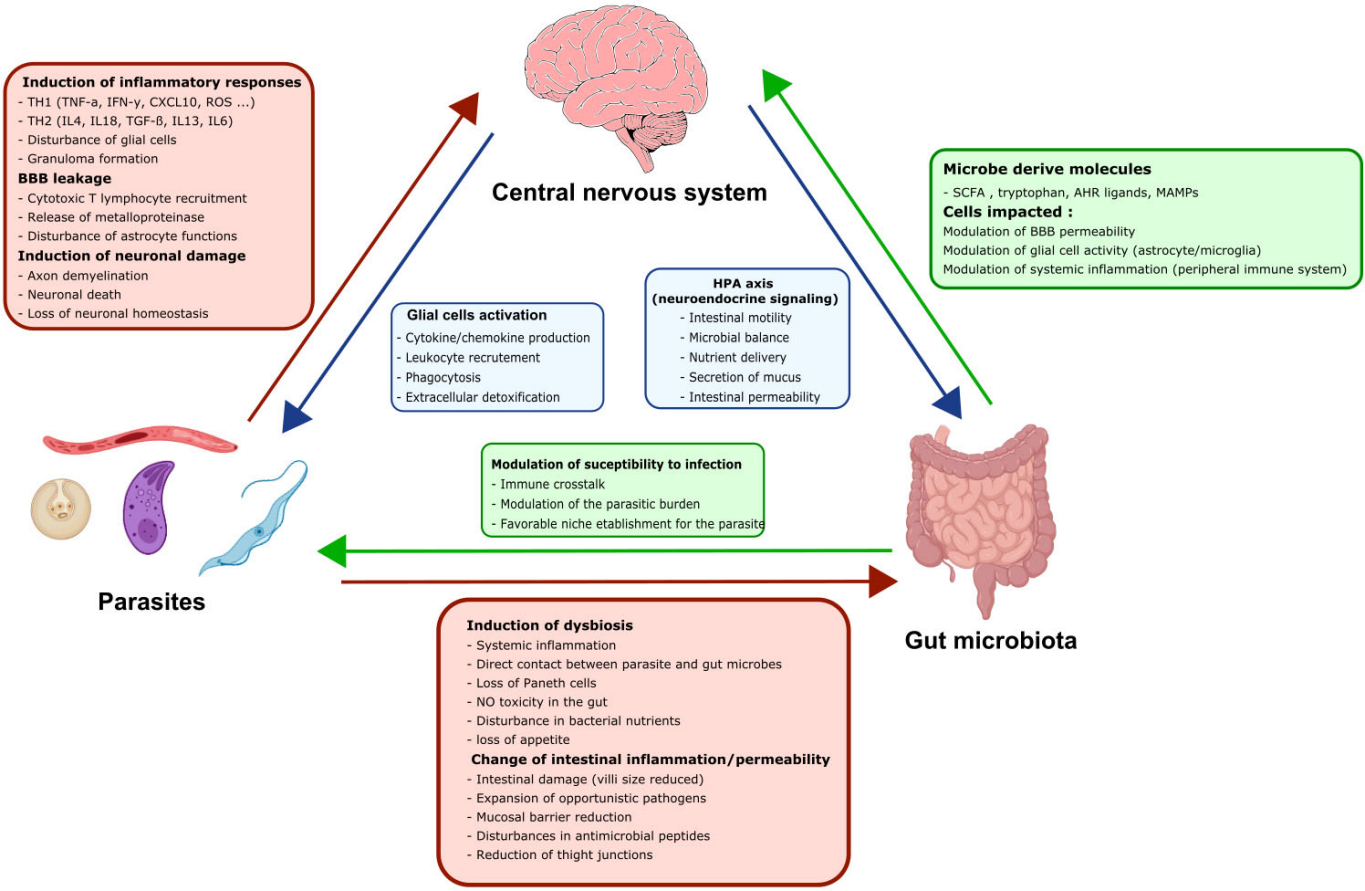
The three-way dialogue between parasites, the gut microbiota, and glial cells. A complex bidirectional dialogue exists between the gut microbiota, the CNS and the parasite. Green boxes and arrows summarize gut microbiota interactions with the CNS and the parasites. Red boxes and arrows represent parasites interactions with the CNS and the gut microbiota. Blue boxes and arrows indicate CNS interactions with the gut microbiota and the parasites.

It has been suggested that a eubiotic GM helps to maintain and/or restore homeostasis of the BBB and to prevent inflammatory processes in the brain. Various signaling molecules produced by the GM enable microbes to communicate with the host’s neuro-immune system, and cytokines are produced by the host in response to microbes (121). Most of these molecules act on glial cells (**Figures 4**, **5**) and might modulate inflammation. Indeed, short-chain fatty acids (SCFAs) can influence the gut–brain axis *via* various mechanisms, including epigenetic modifications. SCFAs can inhibit the TLR4/TBK1/NF-κB/TNF-α pathway in astrocytes and microglial cells by inhibiting histone deacetylase, which results in downregulation of pro-inflammatory cytokine/chemokines production (20, 115, 124–127). Acetate can cross the BBB, and butyrate can restore the barrier’s intactness. SCFAs can also reverse dysfunctional microglial phenotypes in GF mice (82). Interestingly, aryl hydrocarbon receptor agonists inhibit VEGF-ß production and stimulate TGF-α production by the microglia and thereby limit the astrocyte’s inflammatory response, disturbance of the BBB, and the infiltration of leukocytes into the CNS (116, 117) (**Figure 5**).

Furthermore, many researchers have reported on the GM’s influence through the synthesis of various neurotransmitters and neuromodulators with crucial roles in gut-brain communication (**Figure 5**). Indeed, some bacteria are able to produce amino acids like gamma-aminobutyric acid (the main inhibitory neurotransmitter in the CNS). The GM can also regulate the level of serotonin, which has central roles in anxiety and depression and mediates changes in hippocampal levels of 5-hydroxyindoleacetic acid and brain-derived neurotrophic factor and plasma levels of tryptophan (118). The GM can notably control neurotransmitter production through the regulation of glutamate, which can be neurotoxic at high levels (119, 128, 129). Recently, bacterial-surface-derived compounds like peptidoglycans have emerged as potential key regulators of GM–brain interactions (130).

Several strategies can be used to restore the GM, such as (i) the administration of specific nutrients that promote the growth of certain bacterial species (i.e. prebiotics), (ii) the introduction or expansion of "beneficial" bacteria species (i.e. probiotics), and (iii) the wholesale or selective transplantation of a donor GM (i.e. fecal transplantation) (57). Many probiotics reportedly exhibit anti-inflammatory properties, notably in the context of inflammatory bowel diseases (122, 131). Several probiotics have been shown to communicate with the brain and influence behavior through vagal nerve signaling. Promising results have also been reported in the context of autoimmune neurodegenerative diseases like Parkinson's disease and Alzheimer's disease. *Lactobacillus reuteri* is able to metabolize tryptophan into indoles, which bind to the aryl hydrocarbon receptor expressed by astrocytes and microglia; this modulates the production of pro-inflammatory chemokine/cytokines and the cells’ ability to respond to lipopolysaccharide by limiting NFκB translocation (90, 117, 132–134) (**Figure 4**). Probiotics might also interact with enteroendocrine cells, i.e. sensory cells that form synapses with vagal afferents (neuropodia) (135). Lastly, we have shown that some probiotics exhibit anti-inflammatory properties *via* the interaction between peptidoglycan and NOD2 (136). We speculate that probiotic-derived peptidoglycans might constitute a novel means of treating neuro-inflammation.

Along with antiparasitic drugs, the value of fecal transplants and probiotics was recently evaluated in the context of infections by protozoans (such as *Giardia duodenalis*, *Cryptosporidium parvum*, *Eimeria tenella*) and nematodes (such as *Toxocara canis* and *Strongyloides venezuelensis*) (58). Fecal transplantation appears to be the most effective way of restoring the GM and combating neuroinflammatory diseases but is used on critical cases only. A better understanding of the molecular crosstalk between the GM and the brain should lead to the development of novel therapies for CNS inflammation caused by parasite infections.

## Conclusion

The brain inflammation processes induced during parasite infections are not well understood and cannot easily be treated. Therefore, any treatment that can reduce this neuroinflammation will have a considerable public health impact. In this context, strategies that target the GM (notably by the development of food supplements able to regulate acute, harmful inflammation in particular and/or neuroinflammatory diseases in general) should be taken into consideration, along with antiparasite drugs.

## Author contributions

SP designed, revised and supervised the work. JA reviewed the literature, created the figures, and wrote the manuscript. IL and CG participated to the analysis, drafting and revising the manuscript. All authors contributed to the article and approved the submitted version.
