# Supplementary material for: Parasite infections, neuroinflammation, and potential contributions of gut microbiota
Source: Front Immunol. 2022 Dec 8;13:1024998. doi: 10.3389/fimmu.2022.1024998 (PMC9772015; doi:10.3389/fimmu.2022.1024998)
Supplement: Supplementary file 1 [file DataSheet_1.pdf]

	Disease	Parasite	Risk population	Endemic region	Cases or deaths/year	CNS disorder triggered	Cytokines involved	Immune cells involved	References
**Acute neuroinflammation**	**Cerebral malaria**	Plasmodium<br>*falciparum*<br>(Protozoan)	Immunodeficient people<br>Children under 5 years<br>Pregnant women<br>Non-endemic population	Sub-saharan Africa, Latin America, South Est of Asia	435,000 deaths/year	Paralysis<br>Seizures<br>Coma<br>Death	IP10<br>TNF-a<br>IL-6<br>IFN-y	Astrocytes<br>Microglia<br>LtCD8+	(24,28,25)
	**African tryponozomiasis**	Trypanozoma *brucei rhodesiense*<br>(Protozoan)<br><br>Trypanosoma *brucei gambiense*<br>*(*Protozoan*)*	15 and 45 years and living in remote rural areas are considered especially vulnerable	Democratic Republic of the Congo<br>Angola<br>Sudan<br>Republic of Congo<br>Central African Republic	977 cases/year but this is an underestimate because the disease is mostly found in rural communities in the endemic areas	Leukoencephalitis<br>Headache<br>Personality changes<br>Daytime somnolence<br>Sensory motor and Psychiatric disorders<br>Death	IFN-y<br>TNF-a<br>IL-1ß<br>IFNa/b<br>CXCL10	Proliferation of astrocytes<br>Hypertrophy of astrocytes<br>Activation of microglia<br>Hypertrophy of Microglia<br>Formation of microglia nodules<br>T lymphocytes	(12, 10, 11)
**Chronic neuroinflammation**	**Neurocysticercosis**	Taenia *solium*<br>(Cestode)	Children and women from infancy to old age, with a peak incidence at 20–50 years of age	Latin America<br>Africa<br>Asia	Approximately 370,710 individuals were infected with T. solium cysticercosis worldwide, resulting in over 28,000 deaths	Seizures<br>Epilepsy<br>Focal neurological deficits<br>Elevated intracranial pressure<br>Cognitive decline	TNF-a<br>IFN-y<br>IL-1ß	Lymphocyte TH1<br>Astrocytes<br>Microglia<br>Neutrophils<br>Eosinophils<br>Monocytes	(49, 46)
	**Neuroschistomiasis**	Schistosoma *mansoni*<br>(Trematode)<br><br>Schistosoma *haematobium*<br>*(Trematode)*	Non-endemic population<br>Immunodeficient people<br>young people and adults	Tropics and subtropics	Over 200 million people are infected by the parasite but only 4% developed neuroschistosomiasis	Headache<br>Visual disturbances<br>Delirium<br>Seizures<br>Motor impairment<br>Ataxia<br>Encephalopathy	TNF-a<br>IFN-y<br>IL-1ß	Lymphocyte TH1<br>Astrocytes<br>Microglia<br>Neutrophils<br>Eosinophils<br>Monocytes	(12, 54, 11)
	**Neurotoxoplasmosis**	Toxoplasma *gondii*<br>*(Protozoan)*	Pregnant women<br>immunodieficient people	Worldwide	1/3 of the population is infected (2–3 billion people worldwide)	Headache<br>Altered mental status<br>Seizures<br>Focal neurologic deficits Hemiparesis<br>Ataxia<br>Cranial nerve palsies<br>Meningoencephalitis	IFN-α<br>IFN-β<br>IFN-y<br>CXCL10<br>IL-12<br>IL-6	Monocytes<br>Dendritic cells<br>Astrocytes<br>Microglia	(12, 11, 38)
