# Supplementary material for: Parasite infections, neuroinflammation, and potential contributions of gut microbiota
Source: Front Immunol. 2022 Dec 8;13:1024998. doi: 10.3389/fimmu.2022.1024998 (PMC9772015; doi:10.3389/fimmu.2022.1024998)
Supplement: Supplementary file 2 [file DataSheet_2.pdf]

**Neurological symptoms**	**Cerebral malaria**	**Cerebral trypanosomiasis**	**Neurocysticercosis**	**Neurotoxoplasmosis**	**Cerebral Schistosomiasis**
Cerebral edema					
Microhemorrhages					
Cerebral necrosis					
Neurodegeneration					
Axons demyelination					
Cognitive trouble					
Prostration					
Convulsion					
Impaired consiousness					
Visual impairment					
Epilepsy					
Leukoencephalitis					
Mental/Psychiatric disturbances (Demantia, depression, agitation, mania, irritability, hallucination, lack of attention, confusion, mental torpor)					
Motor disorder (Ataxia, central hypotonia, difficulty with balance)					
Sensory disoder					
Insomnia (Daytime somnolence)					
Meningoencephalitis					
Hyperpathia					
Extrapyramidal symptoms					
Parenchymal calcified lesions					
Hydrocephalus					
Headache					
Language disturbances (Aphasia)					
Hemiparesie					
Lethargy					
